# Association between early response of alpha-fetoprotein and treatment efficacy of systemic therapy for advanced hepatocellular carcinoma: A multicenter cohort study from China

**DOI:** 10.3389/fonc.2022.1094104

**Published:** 2023-01-04

**Authors:** Gang Hou, Bo Liu, Zhong-Qi Fan, Chao Li, Jian-Ping Zhang, Yan-Hui Guo, Ru-Yi Zhang, Yi Zheng, Hong Zhu, Nan-Ya Wang

**Affiliations:** ^1^ Cancer Center, First Hospital of Jilin University, Changchun, Jilin, China; ^2^ Department of Hepatobiliary and Pancreatic Surgery, General Surgery Center, First Hospital of Jilin University, Changchun, Jilin, China; ^3^ Department of Hepatobiliary Surgery, Eastern Hepatobiliary Surgery Hospital, Second Military Medical University, Shanghai, China; ^4^ Health Examination Center, Changchun Central Hospital, Changchun, Jilin, China; ^5^ Department of hematology and oncology, Meihekou Central Hospital, Meihekou Jilin, China; ^6^ Department of Medical Oncology, Key Laboratory of Cancer Prevention and Intervention, The First Affiliated Hospital, Zhejiang University School of Medicine, Ministry of Education, Hangzhou, China; ^7^ Department of Medical Oncology, The First Affiliated Hospital, Zhejiang University School of Medicine, Hangzhou, China; ^8^ Department of Medical Oncology, The First Affiliated Hospital of Soochow University, Suzhou, Jiangsu, China

**Keywords:** hepatocellular carcinoma, AFP response, systemic therapy, targeted therapy, immune-based combination therapy

## Abstract

**Background:**

Alpha-fetoprotein (AFP) is a well-identified biomarker in hepatocellular carcinoma (HCC). However, only limited AFP-related studies have evaluated its early response to systemic therapy. This study was performed with the aim of assessing the value of early AFP response in predicting overall survival (OS) and progression-free survival (PFS) in advanced HCC patients receiving systemic therapy.

**Methods:**

This cohort study included HCC patients with baseline AFP ≥ 200 ng/ml and no prior treatment history. A > 20% decline in the serum AFP level from baseline to the first follow-up (i.e., 4~6 weeks after treatment) was defined as an early AFP response. Patient demographic information, clinical characteristics, radiological response, and survival rates were compared between patients with early AFP response and patients without early AFP response. We further utilized multivariate Cox regression to seek characteristics related to OS and PFS.

**Results:**

Among 154 patients, 69 patients (44.8%) showed an early AFP response. The disease control rate (76.8 *vs*. 54.1%; *P* = 0.003) and objective response rate (38.4 *vs*. 11.8%; *P* = 0.001) were significantly higher in patients with an early AFP response. By performing multivariate analysis, early AFP response remained a prognostic factor for longer PFS (HR 0.546; 95% CI 0.371-0.804; *P* = 0.002) and longer OS (HR 0.529; 95% CI 0.335-0.834; *P* = 0.006).

**Conclusion:**

An early AFP response is correlated with longer overall survival and progression-free survival for advanced HCC patients receiving systemic therapy. Moreover, an early AFP response is an independent prognostic factor for longer OS and PFS.

## 1 Introduction

Hepatocellular carcinoma (HCC) is a prevalent malignancy derived from liver cells and poses a tremendous threat to global health (1). Most patients are already in advanced stages when first diagnosed (2). Patients diagnosed with advanced HCC are not eligible for curative surgery. Systemic therapy is the main treatment for patients with advanced HCC. Recently, with the emergence of targeted agents and immune-based combination therapy, the systemic therapeutic options for HCC have expanded (2–5). Sorafenib, lenvatinib, and atezolizumab combined with bevacizumab have been recommended as first-line systemic therapies (6). However, there are several limitations. The clinical benefit varies widely among patients. Some patients have long-term benefits, while others develop primary resistance. Adverse reactions also limit the clinical application. There is an urgent need to find better biomarkers to assess the treatment response in the early stage.

Serum biomarkers are noninvasive, labor-saving, and inexpensive tools for disease prediction, diagnosis, and monitoring. Alpha-fetoprotein (AFP) is a glycoprotein that is widely used in HCC detection, screening, monitoring, and prognosis evaluation (7). Almost 70% of HCC patients show an elevated AFP level in their serum (8). Overexpression of baseline AFP is considered an indicator of poor oncological biology, burden, and survival (9).

In addition to the prognostic impact of baseline biomarker levels, the response of biomarkers to malignant tumors after treatment is increasingly recognized as an effective tool to assess treatment efficacy and predict tumor response (10). Previous studies of patients with HCC have demonstrated early AFP response in transcatheter arterial chemoembolization, chemotherapy, and radiotherapy (3, 11–29). However, due to the limited published data on AFP in newly developed immune-based combination therapy and targeted agents, a small number of studies have assessed the value of early AFP response in advanced HCC patients receiving systemic therapy.

The present study was performed with the aim of investigating whether an early AFP response is associated with systemic therapy in advanced HCC patients by utilizing a multicenter database. We aimed to provide clinicians with data on treatment efficacy monitoring and planning for decision-making.

## 2 Patients and methods

### 2.1 Patient selection

We conducted this multicenter study between May 2018 and May 2021 in seven hospitals. These seven medical centers were the First Hospital of Jilin University, Eastern Hepatobiliary Surgery Hospital, Meihekou Central Hospital, Changchun Central Hospital, The First Affiliated Hospital, Zhejiang University School of Medicine, and the First Affiliated Hospital of Soochow University. We retrospectively analyzed 450 HCC patients receiving lenvatinib, sorafenib, or any immune-based combination therapy (e.g., sintilimab, camrelizumab, toripalimab, et al). Sorafenib, lenvatinib, apatinib, and regorafenib are combined with immunotherapy. We included advanced HCC patients with a baseline AFP level ≥ 200 ng/ml and a duration of medical interventions ≥ 1 month. All patients signed informed consent during hospital admission. The study was conducted in accordance with the Declaration of Helsinki and the Ethical Guidelines for Clinical Studies. The study was approved by the Institutional Review Board of the First Hospital of Jilin University (IRB number 032-06).

### 2.2 Baseline characteristics

The baseline characteristics included sex, age, comorbidities, Eastern Cooperative Oncology Group (ECOG) performance status, etiology of liver disease, cirrhosis, portal hypertension, international normalized ratio, total bilirubin, albumin-bilirubin (ALBI) grade, alanine transaminase, aspartate transaminase, albumin, and Child-Pugh grade. Comorbidities included hypertension, diabetes mellitus, cardiovascular disease, chronic obstructive pulmonary disease, and renal dysfunction. Portal hypertension was defined as the presence of either splenomegaly with a decreased platelet count (≤ 100 × 10^9^/L) or esophageal varicose veins. Tumor-related characteristics included baseline AFP, maximum tumor size, tumor number, macrovascular invasion, and extrahepatic spread.

### 2.3 Patient follow-up

The follow-up strategies were consistent in all participating hospitals. Our strategies included detecting the concentration of serum AFP and performing contrast-enhanced computed tomography (CT) or magnetic resonance imaging (MRI) on a regular basis. The first follow-up evaluation was performed 4~6 weeks after treatment. After the first follow-up, the strategies were performed every 2-3 months. As HCC progression was suspected, contrast-enhanced CT or MRI, chest CT, bone scan, or positron emission tomography were performed as clinically indicated. Our last follow-up was concluded on May 30, 2021.

### 2.4 Biomarker response of serum AFP

Patients with baseline AFP ≥ 200 ng/ml were enrolled in the present study. The first follow-up of AFP was performed 4 to 6 weeks after treatment. In our study, we defined patients with a > 20% decrease in serum AFP level at the first follow-up compared with baseline as having an early AFP response. Correspondingly, patients whose serum AFP levels failed to reach the abovementioned levels were defined as no AFP responders.

### 2.5 Clinical outcomes

The primary clinical outcomes of this study were overall survival (OS) and progression-free survival (PFS). PFS refers to the time from the start of treatment to tumor progression or death. OS refers to the interval from study initiation to either the date of death or the date of the last follow-up. We used enhanced CT or MRI to perform radiological assessment in accordance with RECIST v1.1 criteria. The objective response rate (ORR) is the total proportion of patients with complete response (CR) or partial response (PR). The disease control rate (DCR) is the sum of CR, PR, and stable disease (SD).

### 2.6 Statistical analysis

Statistical analyses were performed using SPSS software version 25.0 (SPSS, Chicago, IL, USA). The patient characteristics between the early AFP response and no response groups were compared using the χ2 test for continuous variables or Fisher’s exact test for categorical variables. Early AFP response was defined as a > 20% decrease in serum AFP levels at the first follow-up. Those patients who died before the first follow-up were excluded. The OS and PFS rates were calculated and compared using the Kaplan-Meier method generated by the log-rank test. Univariate and multivariate Cox regression analyses were performed to identify independent predictors associated with poor OS and PFS, with hazard ratios (HRs) and 95 percent confidence interval (95% CIs). Those variables significant at *P* < 0.1 in the univariable analyses were entered into the multivariable competing-risks regression models. A two-tailed *P* value < 0.05 was considered to be statistically significant.

## 3 Results

### 3.1 Clinical characteristics

**Figure 1:**
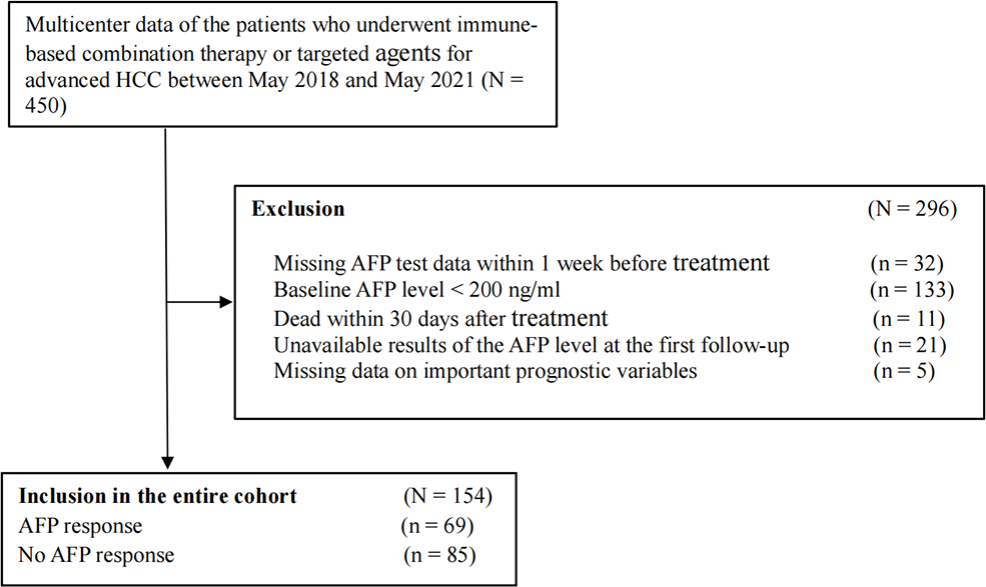
Flow chart of the cohort.

A total of 450 patients with advanced HCC were screened. We excluded patients whose missing AFP data within 1 week before treatment (n = 32), baseline AFP level < 200 ng/ml (n = 133), death within 30 days after treatment (n = 11), unavailable AFP level results at the first follow-up (n = 21), and missing data on important prognostic variables (n = 5). Ultimately, 154 patients who met the inclusion criteria were enrolled in this retrospective cohort study (**Figure 1**). According to the definition of early AFP response, we divided all patients into two groups: 69 (44.8%) patients were in the early AFP response group, and 85 (55.2%) were in the no AFP response group. Demographic and tumor-related characteristics were virtually balanced in the two groups. The median follow-up time is 10.7 months (range, 1.9 to 31.6 months). 23 (15.8%) patients had a dose reduction and 9 (6.2%) had a treatment interruption due to AEs. More detailed information is shown in **Table 1**.

**Table 1:** Comparisons of the patients’ baseline characteristics.

Variables	Total (N=154)	Early AFP Response (N=69)	No AFP Response (N=85)	*P*
**Treatment**				
**Lenvatinib**	64 (41.6)	25 (39.1)	39 (60.9)	0.125
**sorafenib**	35 (22.7)	12 (34.3)	23 (65.7)	
**sintilimab combination therapy**	28 (18.2)	17 (60.7)	11 (39.3)	
**camrelizumab combination therapy**	18 (11.7)	11 (61.1)	7 (38.9)	
**toripalimab combination therapy**	9 (5.8)	4 (44.4)	5 (55.6)	
**Sex, male**	136 (88.3)	59 (43.4)	77 (56.6)	0.329
**Age > 60 years old**	52 (33.8)	24 (46.2)	28 (53.8)	0.810
**Comorbidities**	32 (20.8)	14 (43.8)	18 (56.2)	0.893
**ECOG performance status**				
**0**	48 (31.2)	18 (37.6)	30 (62.4)	0.220
**1-2**	106 (68.8)	51 (48.1)	55 (51.9)	
**Etiology of liver disease**				
**HBV (+) and/or HCV (+)**	109 (70.8)	53 (48.6)	56 (51.4)	0.138
**Others**	45 (29.2)	16 (35.6)	29 (64.4)	
**Cirrhosis**	117 (76)	56 (47.9)	61 (52.1)	0.175
**Portal hypertension**	55 (35.7)	24 (43.6)	31 (56.4)	0.828
Baseline AFP, ng/ml*	1210.0 (668.1,5305.8)	1000.0 (647.7,4651.0)	2000.0 (681.8,10809.9)	0.511
INR,s*	1.04 (0.98,1.12)	1.03 (0.98,1.12)	1.04 (0.99,1.14)	0.921
Total bilirubin, μmol/L*	20.0 (13.0,44.0)	19.0 (11.0,42.0)	20.0 (14.0,45.0)	0.610
Alanine transaminase, U/L*	30.0 (20.6,49.7)	30.0 (21.0,47.9)	32.4 (20.0,53.4)	0.528
Aspartate transaminase, U/L*	42.0 (27.0,66.0)	40.0 (25.6,66.4)	43.5 (28.5,63.8)	0.778
Albumin, g/L*	39.2 (34.8,41.6)	39.6 (36.6,42.1)	38.7 (34.0,40.8)	0.763
**ALBI grade**				
**ALBI grade 1**	60 (39.5)	30 (50.0)	30 (50.0)	0.300
**ALBI grade 2**	94 (61.0)	39 (41.5)	55 (58.5)	
**Child-Pugh grade**				
**Child-Pugh grade A**	126 (81.8)	60 (47.6)	66 (52.4)	0.136
**Child-Pugh grade B**	28 (18.2)	9 (32.1)	19 (67.9)	
**Maximum tumor size > 5.0 cm**	104 (67.5)	48 (46.2)	56 (53.8)	0.627
**Multiple tumors**	104 (67.5)	46 (44.2)	58 (55.8)	0.836
**Gross vascular invasion**	53 (34.4)	24 (45.3)	29 (54.7)	0.931
**Extrahepatic spread**	78 (50.6)	31 (39.7)	47 (60.3)	0.201

* Values are the median and interquartile range.

Abbreviations: AFP, alpha-fetoprotein; ALBI, albumin-bilirubin; ECOG, Eastern Cooperative Oncology Group; HBV, hepatitis B virus; HCV, hepatitis C virus; INR, international normalized ratio.

### 3.2 Correlation of early AFP response with radiologic response

As shown in **Table 2**, we assessed the treatment efficacy in the two groups by RECIST ver1.1. In patients with an early AFP response, 1 (1.4%) patient experienced CR, and 23 (33.3%) patients experienced PR. No patients underwent CR and only 10 (11.8%) patients underwent PR in the group without an early AFP response. Regarding the SD rate, a difference between the two groups was not apparent: 42.0% in the group with an early AFP response and 42.4% in the group without an early AFP response. The PD rate in the early AFP response group was 23.2%, which was lower than the 45.9% in the no AFP response group. Of note, patients in the group with an early AFP response had a higher ORR (38.4% *vs*. 11.8%, *P* = 0.001) and DCR (76.8% *vs*. 54.1%, *P* = 0.003) than the group without an early AFP response.

**Table 2:** Comparison of the efficacy between the patients with and without early AFP response.

	Total (N=154)	Early AFP Response (N=69)	No AFP Response (N=85)	*P*
**RECIST v1.1**				
**Complete response (CR)**	1 (0.6)	1 (1.4)	0 (0)	0.002
**Partial response (PR)**	33 (21.4)	23 (33.3)	10 (11.8)	
**Stable disease (SD)**	65 (42.2)	29 (42.0)	36 (42.4)	
**Progressive disease (PD)**	55 (35.7)	16 (23.2)	39 (45.9)	
**Objective response rate (ORR)**	34 (22.0)	24 (38.4)	10 (11.8)	0.001
**Disease control rate (DCR)**	99 (64.3)	53 (76.8)	46 (54.1)	0.003

### 3.3 Survival analyses of OS and PFS

**Figure 2:**
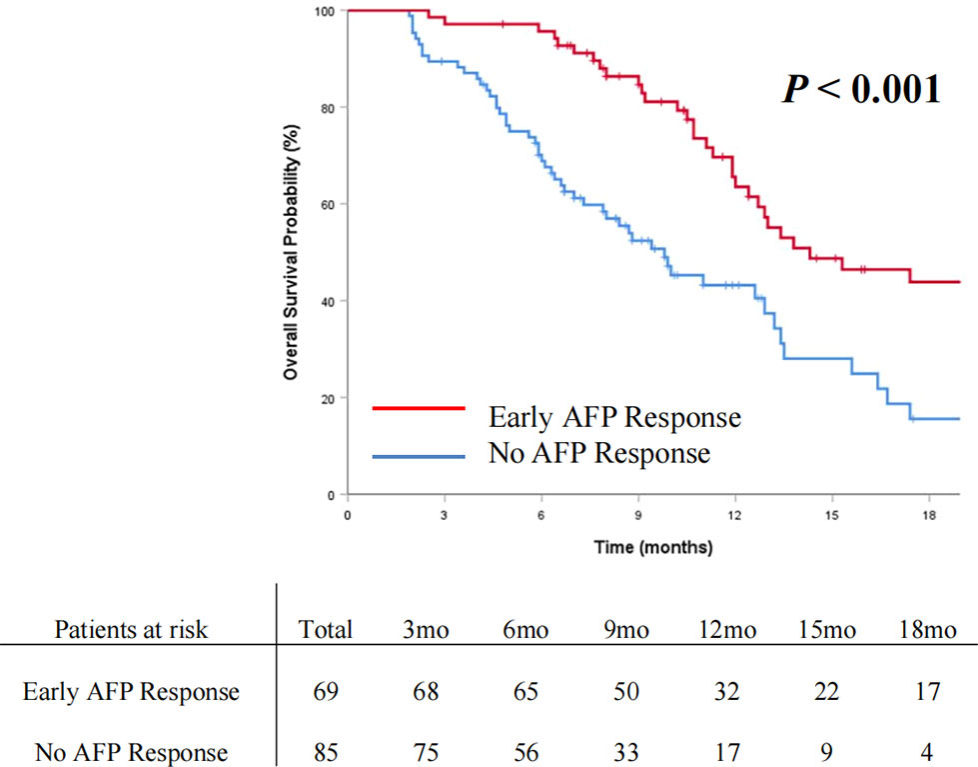
Cumulative incidence of overall survival curve comparisons between the patients with early AFP response and no AFP response in the entire cohort.

**Figure 3:**
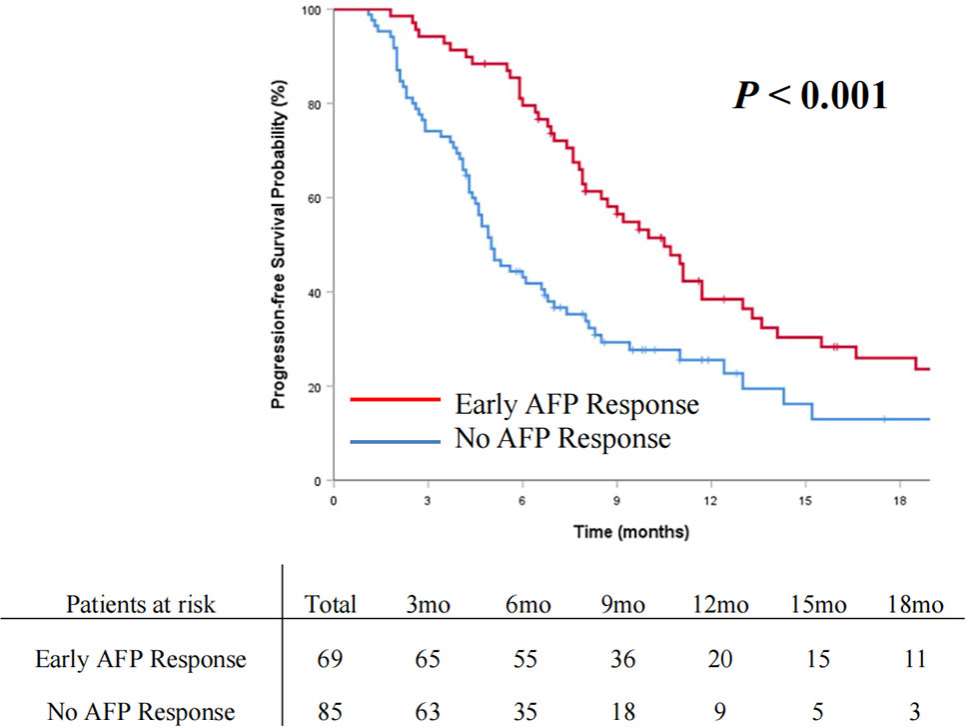
Cumulative incidence of progression-free survival curve comparisons between the patients with early AFP response and no AFP response in the entire cohort.

**Figure 4:**
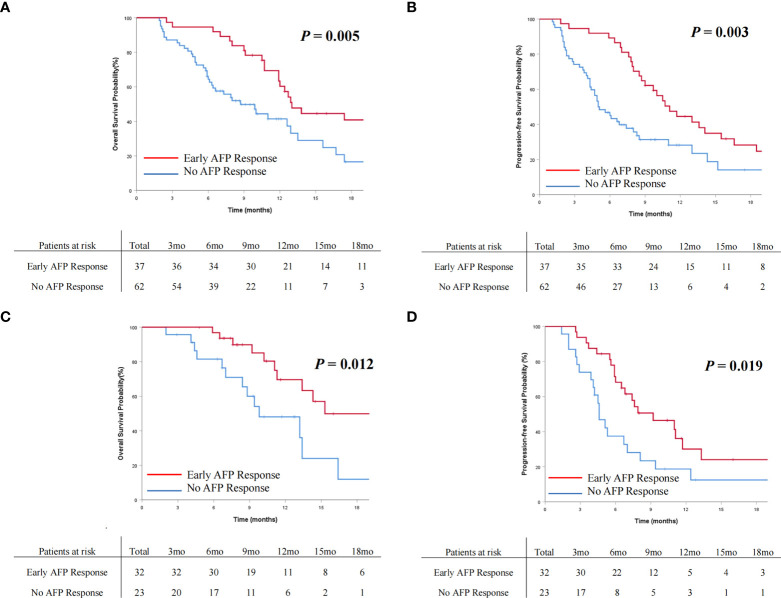
Cumulative incidence of overall survival **(A)** and progression-free survival **(B)** curves comparisons between the patients with AFP response and no AFP response in the targeted agents’ cohort. Cumulative incidence of overall survival **(C)** and progression-free survival **(D)** curves comparisons between the patients with early AFP response and no AFP response in the immune combination targeted therapy cohort.

The OS and PFS curves between the entire cohorts of patients with early AFP response and no AFP response are demonstrated in **Figures 2** and **3**, respectively. Compared with the no AFP response group, the early AFP response group had significantly improved OS (*P* < 0.001) and PFS (*P* < 0.001). Similar results of the OS and PFS rates with a significant difference were shown in the cohorts of patients with the targeted agents (**Figures 4A**, **B**, *P* = 0.005 and *P* = 0.003) and immune combination targeted therapy (**Figures 4C**, **D**, *P* = 0.012 and *P* = 0.019), respectively.

### 3.4 Univariate and multivariate analyses of OS and PFS

In univariate analysis of OS, we found that patients with ECOG performance status of 1 or 2 (HR, 1.949; 95% CI, 1.109-3.426; *P* = 0.020), early AFP response (HR, 0.507; 95% CI, 0.322-0.798; *P* = 0.003), aspartate transaminase (AST) > 40 U/L (HR, 1.748; 95% CI, 1.111-2.750; *P* = 0.016), Child-Pugh B (HR, 2.040; 95% CI, 1.173-3.548; *P* = 0.012), and multiple tumors (HR, 1.546; 95% CI, 0.923-2.588; *P* = 0.098) were likely to achieve longer OS. Adjusting for sex, age, comorbidities, and etiology of cancer, the multivariate analysis indicated that early AFP response (HR, 0.490; 95% CI, 0.308-0.780; *P* = 0.003) and ECOG performance status of 1 or 2 (HR, 2.201; 95% CI, 1.240-3.907; *P* = 0.007) were independent prognostic factors associated with longer OS (**Table 3**).

**Table 3:** Univariate and multivariate Cox regression analyses predicting overall survival.

Variables	Comparison	Univariable Analyses		Multivariate Analyses	
		HR (95% CI)	*P*	HR (95% CI)	*P*
**Sex**	Male *vs.* Female	1.366 (0.628-2.969)	0.432		
**Age**	≤ 60 *vs.* > 60 years	1.402 (0.894-2.199)	0.141		
**Comorbidities**	Yes *vs.* No	1.119 (0.661-1.896)	0.676		
**ECOG performance status**	1/2 *vs.* 0	1.949 (1.109-3.426)	0.020*	2.201 (1.240-3.907)	0.007
**HBV (+) and/or HCV (+)**	Yes *vs.* No	1.487 (0.909-2.434)	0.114		
**Cirrhosis**	Yes *vs.* No	1.187 (0.676-2.086)	0.550		
**Portal hypertension**	Yes *vs.* No	1.214 (0.762-1.936)	0.415		
**Early AFP Response**	Yes *vs.* No	0.507 (0.322-0.798)	0.003*	0.490 (0.308-0.780)	0.003
**Baseline AFP**	> 1000 *vs.* ≤ 1000 ng/ml	1.365 (0.871-2.139)	0.175		
**Alanine transaminase**	> 40 *vs*. ≤ 40 U/L	1.401 (0.897-2.187)	0.138		
**Aspartate transaminase**	> 40 *vs*. ≤ 40 U/L	1.748 (1.111-2.750)	0.016*	1.578 (0.972-2.561)	0.065
**Albumin**	≤ 35 *vs.* > 35 g/L	1.191 (0.753-1.882)	0.455		
**ALBI**	2 *vs*. 1	1.228 (0.783-1.824)	0.371		
**Child-Pugh**	B *vs*. A	2.040 (1.173-3.548)	0.012*	1.660 (0.921-2.990)	0.092
**Maximum tumor size**	> 5.0 cm *vs.* ≤ 5.0 cm	1.126 (0.708-1.791)	0.617		
**Multiple tumors**	Multiple *vs.* Solitary	1.546 (0.923-2.588)	0.098*	1.648 (0.981-2.770)	0.059
**Extrahepatic spread**	Yes *vs.* No	1.184 (0.764-1.836)	0.450		

*Those variables found significant at P < 0.1 in the univariable analyses were entered into the multivariable Cox regression analyses.

AFP, alpha-fetoprotein; CI, confidence interval; ECOG, Eastern Cooperative Oncology Group; HBV, hepatitis B virus; HCV, hepatitis C virus; HR, hazard ratio; ORR, objective response rate; ALBI, albumin-bilirubin; BCLC, Barcelona Clinic Liver Cancer.

The univariate analysis of PFS revealed that the odds of longer OS were high in the patients with ECOG performance status of 1 or 2 (HR, 1.554; 95% CI, 1.036-2.330; *P* = 0.033), early AFP response (HR, 0.538; 95% CI, 0.366-0.791; *P* = 0.002), AST > 40 U/L (HR, 1.865; 95% CI, 1.267-2.745; *P* = 0.002), and multiple tumors (HR, 1.432; 95% CI, 0.937-2.188; *P* = 0.097). By adjusting for sex, age, comorbidities, and etiology of cancer, the multivariate analysis indicated that early AFP response (HR, 0.501; 95% CI, 0.339-0.742; *P* = 0.001), ECOG performance status of 1 or 2 (HR, 1.677; 95% CI, 1.100-2.557; *P* = 0.016) and AST > 40 U/L (HR, 1.933; 95% CI, 1.309-2.855; *P* = 0.001) were independent prognostic factors for longer PFS (**Table 4**).

**Table 4:** Univariate and multivariate Cox regression analyses predicting progression-free survival.

Variables	Comparison	Univariable Analyses		Multivariate Analyses	
		HR (95% CI)	*P*	HR (95% CI)	*P*
**Sex**	Male *vs.* Female	0.954 (0.523-1.742)	0.879		
**Age**	≤ 60 *vs.* > 60 years	1.180 (0.798-1.743)	0.407		
**Comorbidities**	Yes *vs.* No	1.245 (0.786-1.974)	0.350		
**ECOG performance status**	1/2 *vs.* 0	1.554 (1.036-2.330)	0.033*	1.677 (1.100-2.557)	0.016
**HBV (+) and/or HCV (+)**	Yes *vs.* No	1.353 (0.896-2.043)	0.151		
**Cirrhosis**	Yes *vs.* No	1.243 (0.790-1.955)	0.346		
**Portal hypertension**	Yes *vs.* No	1.094 (0.738-1.621)	0.656		
**Early AFP Response**	Yes *vs.* No	0.538 (0.366-0.791)	0.002*	0.501 (0.339-0.742)	0.001
**Baseline AFP**	> 1000 *vs.* ≤ 1000 ng/ml	1.246 (0.853-1.821)	0.255		
**Alanine transaminase**	> 40 *vs*. ≤ 40 U/L	1.302 (0.886-1.195)	0.179		
**Aspartate transaminase**	> 40 *vs*. ≤ 40 U/L	1.865 (1.267-2.745)	0.002*	1.933 (1.309-2.855)	0.001
**Albumin**	≤ 35 *vs.* > 35 g/L	1.302 (0.886-1915)	0.179		
**ALBI**	2 *vs*. 1	1.293 (0.883-1.894)	0.187		
**Child-Pugh**	B *vs*. A	1.232 (0.754-2.035)	0.416		
**Maximum tumor size**	> 5.0 cm *vs.* ≤ 5.0 cm	1.111 (0.744-1.658)	0.607		
**Multiple tumors**	Multiple *vs.* Solitary	1.432 (0.937-2.188)	0.097*	1.409 (0.913-2.175)	0.121
**Extrahepatic spread**	Yes *vs.* No	1.244 (0.855-1.811)	0.254		

*Those variables found significant at P < 0.1 in the univariable analyses were entered into the multivariable Cox regression analyses.

AFP, alpha-fetoprotein; CI, confidence interval; ECOG, Eastern Cooperative Oncology Group; HBV, hepatitis B virus; HCV, hepatitis C virus; HR, hazard ratio; ORR, objective response rate; ALBI, albumin-bilirubin; BCLC, Barcelona Clinic Liver Cancer.

## 4 Discussion

With the therapeutic techniques constantly advancing, immune-based combination therapy and targeted agents have attracted increasing attention as effective systemic therapy methods. Further exploration of biomarkers associated with clinical efficacy and survival benefits is urgent. AFP is an available serum biomarker that is routinely used in clinical practice in many regions of the world for HCC. However, studies utilizing AFP to predict treatment efficacy and survival benefit in systemic treatment for advanced HCC are limited. Our study analyzed advanced HCC patients with high AFP levels receiving systemic therapy. The patients with early AFP response had significantly longer OS (*P* < 0.001) and PFS (*P* < 0.001). By performing multivariate analysis, early AFP response was confirmed as a prognostic factor for both OS and PFS. In the present study, we further verified the role of early AFP response in predicting prognosis for advanced HCC patients receiving systemic therapy, which is consistent with published studies (11, 12, 18, 27).

As a well-identified biomarker, early AFP response has demonstrated incomparable predictive value in both systemic and locoregional therapies for HCC patients. However, these studies assessed early AFP response using different criteria. It is difficult to make a widely recognized, accurate, and clinically significant definition of early AFP response. On the one hand, choosing an appropriate baseline AFP value is crucial for clinicians. Some studies analyzed patients with baseline AFP ≥ 40 ng/ml (30). Some studies chose 20 ng/ml as the baseline (12). This is because AFP is not distinctive and specific for cancer diagnosis: serum AFP may not demonstrate elevated levels in approximately 30-40% of HCC patients, and AFP fluctuations are seen not only in HCC but also in chronic liver diseases caused by cirrhosis or chronic viral hepatitis (7). To rule out the interfering factors mentioned above, our study excluded patients whose AFP levels were positive but low (20-200 ng/mL) and negative (< 20 ng/mL). We also strictly set the baseline AFP level (≥ 200 ng/ml) to ensure higher specificity. For the analysis, a relatively high baseline was chosen to capture real changes related to HCC and minimize the effects of background sources and hospital changes on different tests. On the other hand, selecting a precise time point to measure serum AFP levels is also crucial for clinicians. In a recent study, the first follow-up choice was 7 days after treatment (31). Some studies detected serum AFP levels at 90 days after treatment, and another study was performed at 180 days (32–34). These studies all drew a similar conclusion that early AFP response is independently related to the progression rate and overall survival rate after treatment of HCC. However, considering the half-life of AFP to be 5~7 days, 7 days seems too short, while 90 days or 180 days are too late to start early adjuvant treatments against progression. Therefore, in this analysis, the time period of 4-6 weeks after treatment was selected as the first follow-up.

In the present study, we found that there was a close correlation between early AFP response and imaging findings in most patients. The rates of ORR and DCR in the patients with early AFP response were significantly higher than those in the patients with no AFP response (38.4% *vs*. 11.8%, *P* = 0.001, and 76.8% *vs*. 54.1%, *P* = 0.003). Our results are consistent with many previous studies. Chan et al. reported on the correlation between AFP response and the achievement of imaging response (12). Another study reported that patients with AFP response versus no response had a significantly higher ORR (68.4% *vs*. 7.1%; *P* < 0.001) and DCR (84.2% *vs*. 36.0%; *P* = 0.009) (35). Early AFP response may be considered a supplement or alternative to RECIST v1.1 to systemic therapy for HCC.

For several years, circulating tumor cells, microRNAs or DNAs have been used to predict accurate prediction and early detection of tumor progression. However, sophisticated technology and expensive costs limit their applicability. High AFP secreted by tumor cells is a feature of HCC. Once the tumor has progressed after treatment, more AFP will be produced, and the serum AFP levels will be elevated. The present study provides further proof of this theory. Clinicians may consider changing treatment regimens when tumor progression occurs.

There are some limitations to the present study. First, as a retrospective study, it has its intrinsic defects. Second, in addition to tumor response, changes in AFP may also be affected by cirrhosis or chronic viral hepatitis. Although we set a stricter baseline AFP level (200 ng/ml) to minimize the effects of background sources, further studies with long-term follow-up are still needed. Third, the generalizability of the study might be restricted to the Chinese population since most HCC patients in the present study had a history of HBV infection and related diseases. In contrast, HCC cases in the United States and Europe are mainly caused by HCV infection and overconsumption of alcohol. The study needs to be verified in different populations.

In conclusion, the present study revealed that an early AFP response may be applied as an indicator to predict survival and surveillance progression in patients with advanced HCC receiving targeted agents or immune-based combination therapy. For HCC patients with high AFP levels, an early AFP response is a noninvasive and practical alternative endpoint to assess the long-term tumor prognosis of patients. If an AFP response is not reached in the first follow-up, it is necessary to actively consider strengthening the frequency of examinations or changing the treatment plan in time.

## Data availability statement

The raw data supporting the conclusions of this article will be made available by the authors, without undue reservation.

## Ethics statement

The studies involving human participants were reviewed and approved by The Institutional Review Board of the First Hospital of Jilin University. The patients/participants provided their written informed consent to participate in this study.

## Author contributions

GH and BL contributed equally to this work. Study design: N-YW, GH, and CL. Data collection and acquisition: GH, BL, Z-QF, CL, J-PZ, R-YZ, YZ, HZ, and N-YW. Manuscript preparation: GH and BL. Critical revision: N-YW. Final approval of manuscript: All authors. All authors contributed to the article and approved the submitted version.
